# High intraluminal pressure promotes vascular inflammation via caveolin-1

**DOI:** 10.1038/s41598-021-85476-z

**Published:** 2021-03-15

**Authors:** Danielle L. Michell, Waled A. Shihata, Karen L. Andrews, Nurul Aisha Zainal Abidin, Ann-Maree Jefferis, Amanda K. Sampson, Natalie G. Lumsden, Olivier Huet, Marie-Odile Parat, Garry L. Jennings, Robert G. Parton, Kevin J. Woollard, David M. Kaye, Jaye P. F. Chin-Dusting, Andrew J. Murphy

**Affiliations:** 1 Baker Heart and Diabetes Institute, Melbourne, VIC Australia; 2 Department of Medicine, Monash University, Clayton, VIC Australia; 3 Cardiovascular Disease Program, Biomedicine Discovery Institute, Monash University, Clayton, Australia; 4 Department of Pharmacology, Monash University, Clayton, VIC Australia; 5 Institute for Molecular Bioscience and Centre for Microscopy and Microanalysis, University of Queensland, St Lucia, QLD Australia; 6 School of Pharmacy, University of Queensland, St Lucia, QLD Australia; 7 Centre for Inflammatory Disease, Department of Immunology and Inflammation, Imperial College London, London, UK

**Keywords:** Hypertension, Vascular diseases, Circulation, Inflammation, Physiology

## Abstract

The aetiology and progression of hypertension involves various endogenous systems, such as the renin angiotensin system, the sympathetic nervous system, and endothelial dysfunction. Recent data suggest that vascular inflammation may also play a key role in the pathogenesis of hypertension. This study sought to determine whether high intraluminal pressure results in vascular inflammation. Leukocyte adhesion was assessed in rat carotid arteries exposed to 1 h of high intraluminal pressure. The effect of intraluminal pressure on signaling mechanisms including reactive oxygen species production (ROS), arginase expression, and NFĸB translocation was monitored. 1 h exposure to high intraluminal pressure (120 mmHg) resulted in increased leukocyte adhesion and inflammatory gene expression in rat carotid arteries. High intraluminal pressure also resulted in a downstream signaling cascade of ROS production, arginase expression, and NFĸB translocation. This process was found to be angiotensin II-independent and mediated by the mechanosensor caveolae, as caveolin-1 (*Cav1*)-deficient endothelial cells and mice were protected from pressure-induced vascular inflammatory signaling and leukocyte adhesion. *Cav1* deficiency also resulted in a reduction in pressure-induced glomerular macrophage infiltration in vivo. These findings demonstrate Cav1 is an important mechanosensor in pressure-induced vascular and renal inflammation.

## Introduction

Hypertension remains the single biggest contributor to the global burden of disease and mortality accounting for 9.4 million deaths worldwide each year^1,2^. Specifically, it is a major risk factor for ischaemic cardiovascular disease^3^, chronic kidney disease^4^ and stroke^5^. While there is an abundance of reports on the aetiology and clinical management of raised blood pressure, far less is known about whether pressure itself directly contributes to the pathogenesis of atherosclerosis. Understanding how raised blood pressure causes end organ damage is critical for successful management of this condition.

Vascular endothelial and smooth muscle cell inflammation is a complex and dynamic process involving expression and activation of adhesion molecules, reactive oxygen species (ROS) and innate and adaptive immune cell recruitment and infiltration into these tissues. Significant advances have been achieved in recognising the contributory roles of T cells^6^, B cells^7^ and infiltrating monocytes^8^ in the progression of essential hypertension. Supporting this, there is a positive association between high blood pressure and inflammatory cytokines/chemokines, such as CC chemokine ligand 2 (CCL2; also known as monocyte chemoattractant protein 1 (MCP1))^9,10^. In experimental hypertension models, enhanced expression of adhesion molecules including ICAM-1 and P-selectin has been observed upon stimulation of nuclear factor κB (NF-κB)^11^. Together these pathways aid in the recruitment and adhesion of the immune cells involved in hypertensive-related vascular pathologies.

To date, activation of the renin-angiotensin system and oxidative stress have been the principal explanation for stimulation of inflammatory pathways leading to vascular pathologies. However, the role of elevated intravascular pressure as a perpetuating mechanism has not been explored. Thus, we aimed to explore if induction of high intraluminal pressure (to mimic hypertensive conditions) promoted endothelial inflammation and attraction of circulating leukocytes in rat and mouse models. Understanding the relevance of high intravascular pressure will provide insights on why maintenance of normal blood pressure in those with hypertension is important to prevent hypertension-associated end organ damage.

## Results

### Acute high intraluminal pressure induces endothelial inflammation and leukocyte adhesion

**Figure 1:**
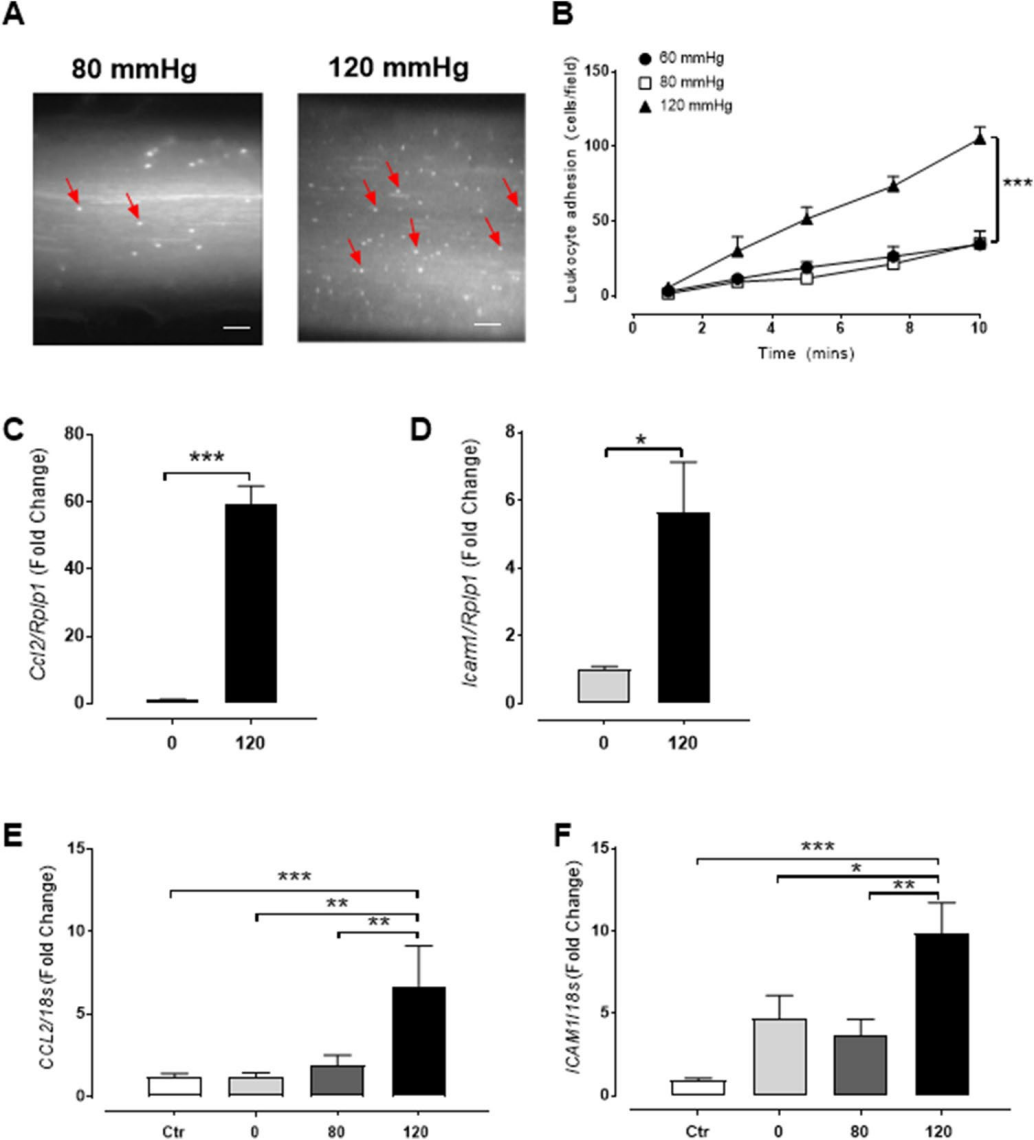
Acute high intraluminal pressure induces vascular endothelial inflammation. (**A-D**) Carotid arteries from 10 week-old Sprague Dawley rats were excised and pressurised (0, 60, 80, 120 mmHg) for 1 h. (**A**) Representative images of dynamic human leukocyte adhesion labelled with DiI in rat carotid arteries (RCA) under fluorescence at 80 and 120 mmHg after 10 min of perfusion. Bar = 200 µm. (**B**) Quantification of leukocytes adhered per field over 10 min (n = 4–5). (**C** and **D**) Gene expression of rat *Ccl2* and *Icam1* relative to *Rplp1* in RCA pressurised at 0 and 120 mmHg for 1 h normalised to 0 mmHg (n = 5–6). (**E,F**) HUVECs pressurised at 0, 80 and 120 mmHg for 1 h and mRNA expression of *CCL2* and *ICAM1* relative to *18 s* was determined and results normalised to an unpressurised control (Ctr; n = 5–10). All results are expressed as mean ± SEM. Data were analysed with a Student’s t-test (**C** and **D**), one-way (**E** and **F**) or two-way ANOVA (**B**) with Bonferroni post hoc test where *p < 0.05, **p < 0.01 and ***p < 0.001.

Given the association with hypertension and vascular inflammation, we first explored if high intraluminal pressure could facilitate the recruitment of leukocytes. This was examined using an ex vivo real-time adhesion assay model under varying pressure. We discovered that leukocyte adhesion over a 10 min perfusion period at physiological sheer stress, was significantly increased in rate carotid artery (RCA) segments pressurised for 1 h at 120 mmHg compared to segments pressurised at low (60 mmHg) and normotensive (80 mmHg) conditions (Fig. 1A-B). Consistent with the enhanced leukocyte adhesion, we observed a significant increase in mRNA expression of endothelial adhesion molecules (intracellular adhesion molecule 1 [*Icam1*], and monocyte chemoattractant protein 1 [MCP1/*Ccl2*]) in arteries pressurised at 120 mmHg compared to unpressurised vessels (Fig. 1C-D). To confirm the role of the endothelium, human umbilical vein endothelial cells (HUVECs) were cultured and pressurised for 1 h. The HUVECs also demonstrated a similar mRNA pattern with increased *CCL2* and *ICAM1* expression at 120 mmHg compared to 80 mmHg or 0 mmHg (Fig. 1E-F). These data indicate that acute high intraluminal pressure induces leukocyte adhesion and endothelial activation.

The haemodynamic forces that blood vessels are subjected to include circumferential wall stretch and fluid shear stress. Increases in pressure directly increase the circumferential stretch, which, as we have shown, increases adhesion. In the experiments described above, vessels were subjected to increased pressure for 1 h without flow (i.e. no shear). To examine the effect of different flow situations during incubation, RCAs were subjected to low shear stress (LSS; 1.67 dyne/cm^2^; 0.1 ml/min) or high shear stress (HSS; 16.7 dyne/cm^2^; 1 ml/min) according to the Hagen-Poiseuille equation for 1 h^12,13^. Adhesion in RCAs subjected to 120 mmHg and LSS was not different to those pressurised to 120 mmHg with no shear (Figure S1A). However, adhesion in vessels under 120 mmHg and HSS was reduced, supporting reports in the literature of the protective effects of HSS^14–16^ and that LSS does not induce inflammation without pressure. In addition, gene expression of *Ccl2* (Figure S1B) showed a similar trend where LSS increased pressure-induced inflammation and HSS blunted it, no change was observed with *Icam1* (Figure S1C).

### Increased production of ROS plays a role in pressure-induced adhesion

**Figure 2:**
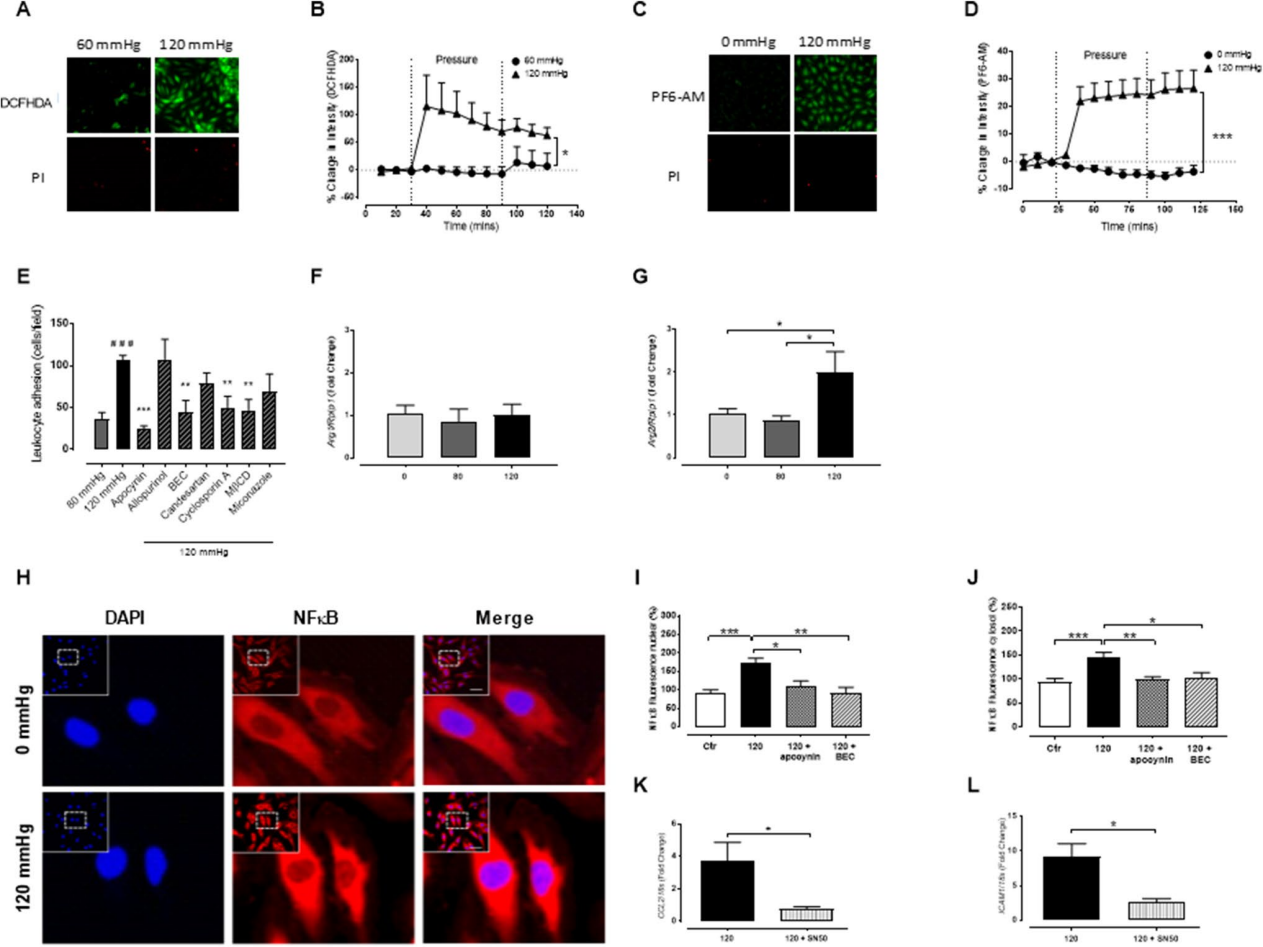
Pressure-induced endothelial activation promotes reactive oxygen species (ROS) production and arginase II via NFĸB-dependent pathways. (**A-D**) Cultured HUVECs were pressurised (0, 60 or 120 mmHg) for 1 h in a flow-through chamber and the % change in intensity of 2′-7′ dichlorodihydrofluorescein diacetate (**A-B**; DCFHDADA; 5 μM; n = 5–7) and peroxyfluor-6 acetoxymethyl ester (**C-D**; PF6-AM; 5 μM; n = 6–7) assessed. Propidium iodide (PI; 5 μM) was also visualized to assess cell death. (**E**) Quantification of leukocyte adhesion (at 10 min) during high pressure (120 mmHg) coupled with either NADPH oxidase inhibitor (apocynin; 3 μM), xanthine oxidase inhibitor (allopurinol; 100 μM), arginase inhibitor (BEC, [S-(2-boroethyl)-L-cysteine], angiotensin receptor 1 antagonist (candesartan; 100 nM), mitochondrial ROS inhibitor (cyclosporine A; 2 μM), 100 μM), cholesterol chelant (methyl-β-cyclodextrin, MβCD; 10 mM) or the cytochrome P450 inhibitor (miconazole; 30 μM) (n = 4–5). (**F** and **G**) Real-time PCR of pressurised carotid arteries (0, 80 and 120 mmHg) was performed to determine the gene expression of arginase I (*ArgI*; n = 3–4) and arginase II (*ArgII*; n = 4–7) isoforms. (**H-J**) HUVECs were seeded onto glass slides, unpressurised (0 mmHg) or pressurised to 120 mmHg and fluorescently probed and imaged in the absence and presence of the NADPH oxidase inhibitor, apocynin (3 µM) or the arginase inhibitor BEC (100 µM) to determine the nuclear (**I**) and cytosolic (**J**) expression of NFĸB (nuclear factor κB, n = 5–11). (**K**) *CCL2* and (**L**) *ICAM1* gene expression in HUVECs pressurised to 120 mmHg was assessed in the presence and absence of the NFκB inhibitor, SN50 (20 µM), which prevents translocation of cytosolic NFκB to the nucleus (n = 5–6). All results are expressed as mean ± SEM. Data were analysed with a Student’s t-test (**E**, **K**, and **L**), a one-way (**F**-**G** and **I**-**J**), or a two-way ANOVA (**B**, **D**) with Bonferroni post hoc test where *p < 0.05, **p < 0.01 and ***p < 0.001.

As reactive oxygen species contribute to hypertension^17^ and inflammation^18^, we assessed whether differences in ROS production may contribute to the increased vascular inflammation observed with pressure. In comparison to the baseline, HUVECs pressurised at 120 mmHg but not 60 mmHg exhibited significant increase in 2′-7′ dichlorodihydrofluorescein diacetate (DCFHDA) intensity (Fig. 2A-B). In addition, using a specific hydrogen peroxide probe, PF6-AM, there was a rapid sustained increase in ROS production in HUVECS pressurised at 120 mmHg (Fig. 2C-D). Furthermore, pressurised RCA incubated with apocynin (3 µM), or the mitochondrial ROS inhibitor cyclosporin A (2 µM), showed reductions in vascular adhesion (Fig. 2E). In contrast, inhibition of ROS production by xanthine oxidase or by cytochrome P450 by allopurinol (100 µM) and miconazole (30 µM), respectively, did not affect leukocyte adhesion (Fig. 2E). Our data suggest that pressure-induced leukocyte adhesion is influenced by ROS production from the mitochondria and NADPH oxidase.

### Angiotensin 1 receptor blockade has no effect on pressure-induced inflammation

To confirm that the inflammatory effects observed were directly induced by high intraluminal pressure and not influenced by the release of angiotensin II (Ang II), we examined the effects of pressurising RCA at 120 mmHg in the presence of the AT1 receptor blocker, candesartan (100 nM; Fig. 2E). Under these conditions, we did not observe statistically significant changes in leukocyte adhesion in comparison to pressure alone suggesting that the pressure-induced adhesion observed is Ang II-independent.

### A role for arginase in pressure-induced inflammation

The enzyme arginase (isoforms I & II) competes with eNOS for the substrate L-arginine, and arginase expression and activity is enhanced in hypertensive animals^19^ and humans^20^. Indeed, adhesion in RCA pressurised to 120 mmHg in the presence of the arginase inhibitor BEC (100 µM) was reduced to similar levels as vessels pressurised to 80 mmHg (Fig. 2E). Gene expression of the arginase II isoform was also increased at 120 mmHg but not 80 mmHg when compared to control while expression of the arginase I isoform was unchanged with pressure (80 & 120 mmHg) (Fig. 2F-G). These data suggest that arginase II expression is increased with pressure and directly contributes to leukocyte adhesion to the endothelium.

### Pressure-induced endothelial activation is NFκB dependent

A number of genes that control cytokine-induced endothelial adhesion molecule expression are driven by the transcription factor, nuclear factor κB (NFκB). Activation of NFκB relies on the degradation of its cytoplasmic inhibitor, IκBα, which allows the unbound NFκB to translocate to the nucleus triggering adhesion molecule protein expression. To examine whether an increase in pressure affects NFκB activity, we treated HUVECs, unpressurised (0 mmHg) or pressurised to 120 mmHg, and fluorescently tracked the cellular location of NFκB by confocal microscopy. HUVECs pressurised at 120 mmHg (1 h) demonstrated increased nuclear and cytosolic NFκB fluorescence (Fig. 2H-J). This effect was diminished by the NADPH oxidase inhibitor, apocynin, as well as the arginase inhibitor, BEC (Fig. 2I-J). Furthermore, the effect of high pressure on adhesion molecule gene expression in HUVECs was reduced in the presence of SN50 (20 µM), which inhibits translocation of cytosolic NFκB to the nucleus (Fig. 2K-L).

### Reduced endothelial integrity enhances pressure-induced inflammation

**Figure 3:**
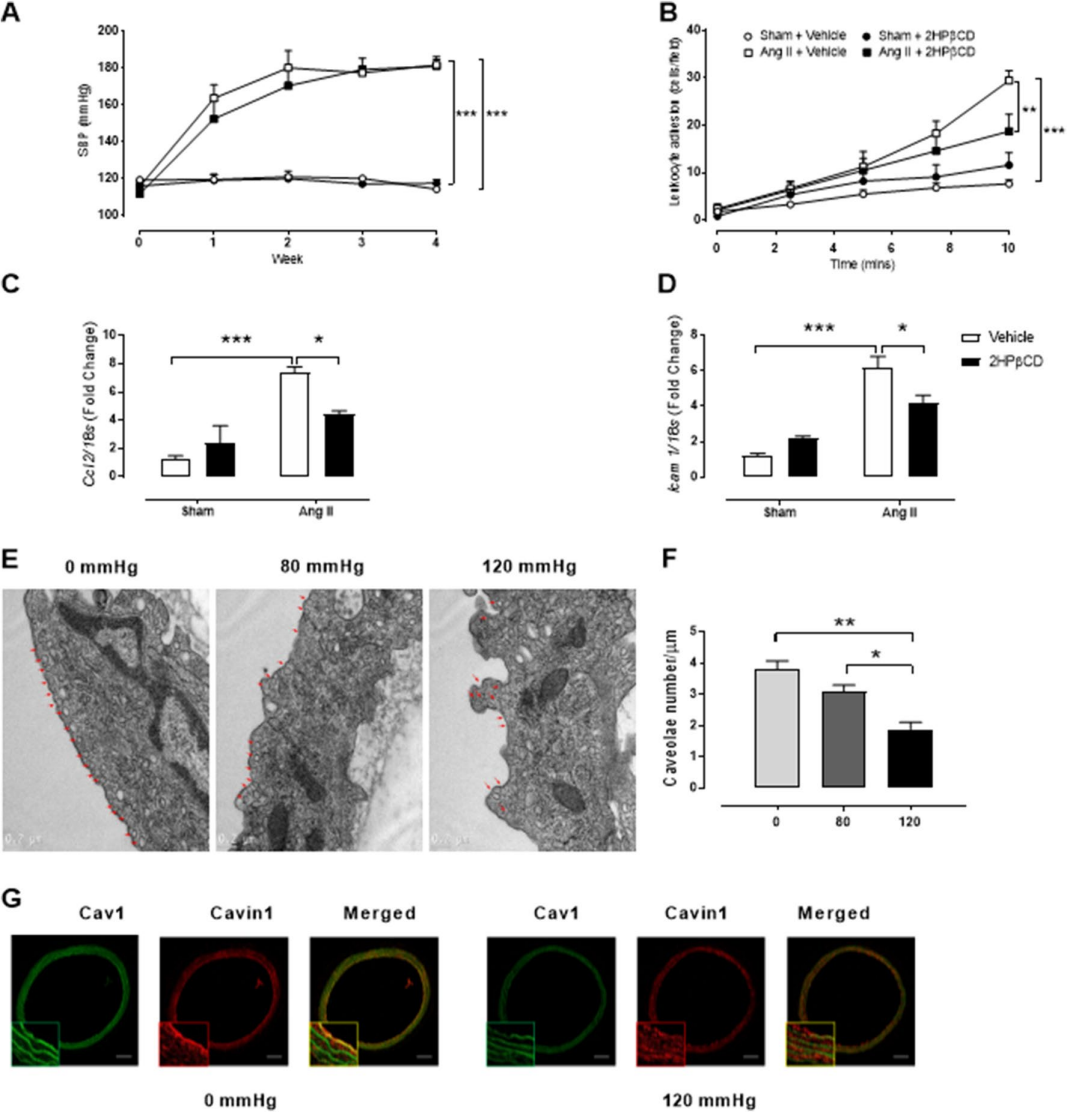
Pressure-induced endothelial inflammation may be via the disruption of caveolae. (**A-B**) 8–10 week-old wild-type C57BL/6 J mice were administered either saline (Sham) or angiotensin II (Ang II; 490 ng/kg/min) via osmotic minipumps over a 4-week period. In the final 2 weeks, mice were subcutaneously treated with saline (vehicle) or 2-hydroxypropyl-β-cyclodextrin (2HPβCD; 2 g/kg body weight) twice a week. (**A**) Systolic blood pressure (SBP) was carried out weekly for the 4-week treatment via tail-cuff plethysmography (n = 8–11). (**B**) Leukocyte adhesion was quantified in aortae harvested from treated mice (n = 6–8). (**C-D**) Real-time PCR was performed to determine the gene expression of *Ccl2* and *Icam1* in collected mouse aortae (n = 4–6). (**E**) Representative ultrathin resin sections of the endothelium of rat carotid arteries after 1 h of pressure (0, 80, 120 mmHg) were examined by electron microscopy (60,000x). Arrows mark caveolae along the plasma membrane. Bar = 0.2 µm. (**F**) Quantification of caveolae detected per μm of plasma membrane (n = 3). (**G**) Distribution of Cav1 and cavin-1 in carotid arteries. Representative confocal immunofluorescence images of Cav1 and cavin-1 in carotid arteries pressurised (0 and 120 mmHg) for 1 h. Scale bar = 20 µm with zoomed images × 3. All results are expressed as mean ± SEM. Data were analysed with a one-way (**F**) or two-way ANOVA (**A-D**) with Bonferroni post hoc test where *p < 0.05, **p < 0.01 and ***p < 0.001.

In order to investigate the impact of membrane integrity on the leukocyte adhesion to the endothelium we assessed the cholesterol chelant methyl-β-cyclodextrin (MβCD), coupled with high pressure (120 mmHg) in RCA. As we found that exposure of RCA to MβCD (10 mM) caused a significant reduction in leukocyte adhesion (Fig. 2E), we aimed to determine if this also occurred in vivo. This was achieved by treating C57BL/6 J mice with Ang II or saline (Sham) via osmotic minipumps for 4 weeks, and in the final 2 weeks intervening with 2-hydroxypropyl-β-cyclodextrin (2HPβCD; 2 g/kg body weight) or vehicle (saline). Ang II-treated mice demonstrated increased blood pressure as measured by tail-cuff plethysmography; however, there was no effect on pressure with 2HPβCD treatment (Fig. 3A). Importantly, mice receiving Ang II treatment demonstrated increased leukocyte adhesion in the aortae compared to sham-treated mice, and this was dampened when coupled with 2HPβCD (Fig. 3B). In addition, inflammatory gene expression markers *Icam1* and *Ccl2* showed a similar trend with increased expression during Ang II treatment, which was dampened when 2HPβCD was added (Figs. 3C-D). These results highlight the importance of endothelial integrity in pressure sensing, which is downstream of increased blood pressure.

### Caveolae play a critical role in pressure-induced inflammation

Of the various mechanosensors that line the plasma membrane, the structural microdomains known as caveolae, are associated with sensing pressure and regulating inflammation^21^. Caveola are 60–80 nm pits enriched in cholesterol and sphingolipids proposed to house both eNOS and NADPH oxidase^22,23^. To examine the consequences of high pressure on caveola density and integrity on the plasma membrane in carotid endothelial cells, we imaged sections of pressurised RCA (1 h) with an electron microscope (Fig. 3E). For each vessel, a minimum of 25 endothelial cells were counted with two fields imaged per cell. Strikingly, high pressure (120 mmHg) reduced the number of caveolae per µm length of membrane when compared to 80 and 0 mmHg (Fig. 3E-F). In addition, morphological changes were evident with a deformation of the endothelial layer in the vessels pressurised to 120 mmHg. The reduction in caveola numbers with pressure was also confirmed with ruthenium red staining (0 mmHg: 100% vs 120 mmHg 43%; Figure S2). A possible explanation for the reduction of caveola number seen in pressurised vessels is the disassembling of caveolae structural proteins caveolin-1 (Cav1) and cavin-1, which have previously been shown to rapidly dissociate when caveolae flatten out in response to stress^24^. Indeed, altered immunofluorescent staining of Cav1 and cavin-1 in pressurised RCA, suggest caveolar disassembly at 120 mmHg (Fig. 3G).

**Figure 4:**
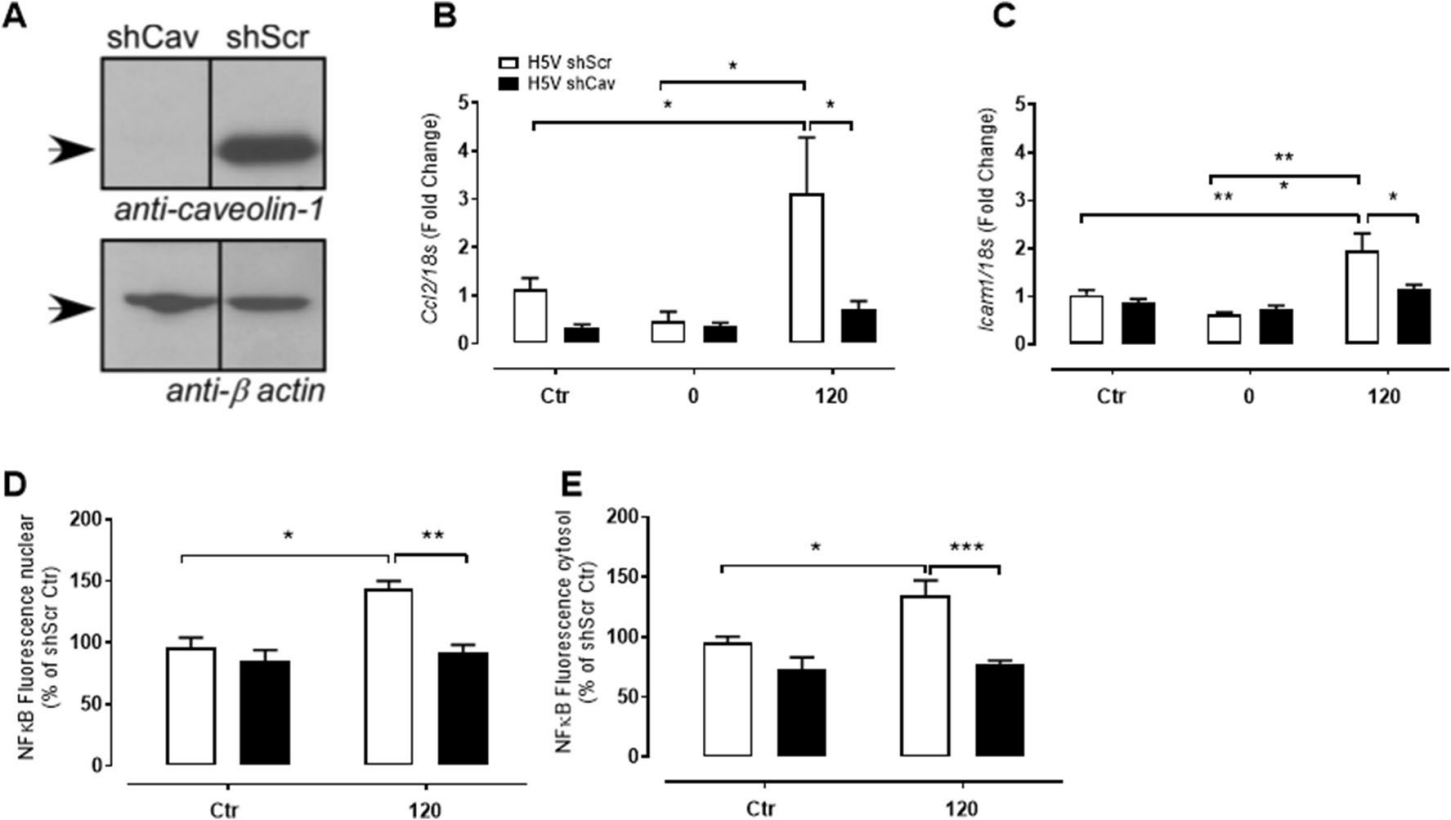
Pressure-induced endothelial activation is caveolin-1 dependent. (**A**) Transformed H5V cells were transfected with either scrambled shRNA (H5V shScr) or *Cav1* shRNA (H5V shCav) (Full blot Figure S3). (**B**) *Ccl2* and (C) *Icam1* gene expression in H5V shScr (white bars) and H5V shCav (black bars) cells that were untreated (Ctr), or pressurised at 0 or 120 mmHg for 1 h (n = 4–6). (**D**) Nuclear and (**E**) cytosolic NFĸB fluorescence (normalized to shScr Ctr) was also quantified in H5V shScr and H5V shCav cells that were unpressurised (Ctr) or pressurised to 120 mmHg (n = 5). All results are expressed as mean ± SEM. Data were analysed with a two-way ANOVA (**B-E**) with Bonferroni post hoc test where *p < 0.05, p < 0.01 and ***p < 0.001.

To observe the role of caveolae in pressure-induced inflammation, H5V cells, derived from mouse embryonic heart endothelium, were transfected with a lentivirus with shRNA to caveolin-1 (H5V shCav, Fig. 4A). H5V shCav were pressurised along with a scrambled shRNA control (H5V shScr) and assessed for inflammatory markers. Interestingly, there was blunted *Ccl2* and *Icam1* gene expression in H5V shCav cells pressurised to 120 mmHg compared to H5V shScr cells under the same pressure (Fig. 4B-C). NFκB expression (immunofluorescence) was also elevated in the nucleus and cytosol of H5V shScr cells pressurised to 120 mmHg and this effect was no longer apparent in H5V shCav cells (Fig. 4D-E). These findings suggest that pressure induces endothelial inflammation via a caveolin-1 dependent mechanism that triggers the NFκB pathway.

### Ang II- and noradrenaline-induced hypertension induces endothelial inflammation and renal macrophage infiltration via caveolin-1

**Figure 5:**
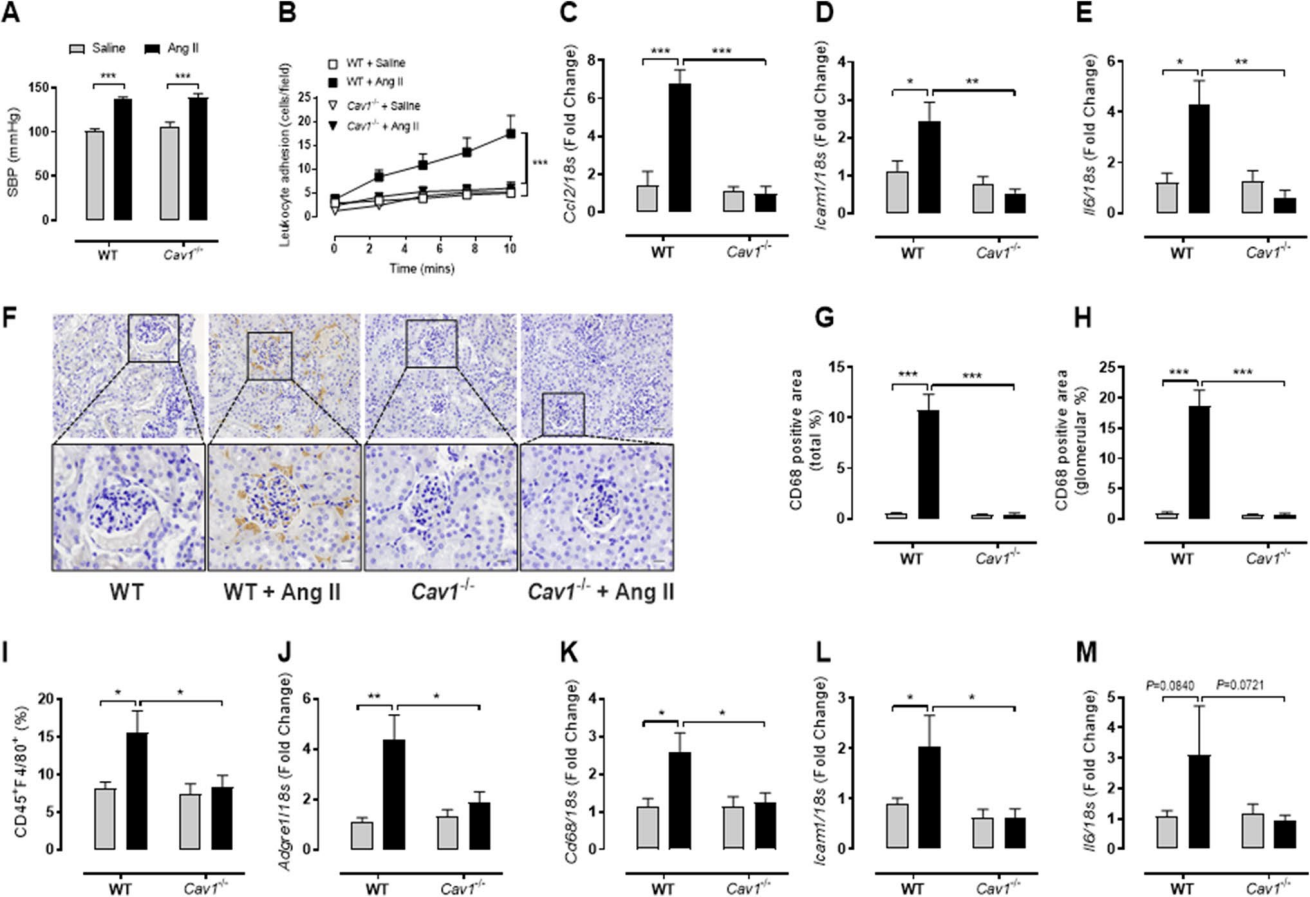
Caveolin-1 is necessary for vascular inflammation and macrophage infiltration in Ang II-induced hypertension. Minipumps containing saline or angiotensin II (Ang II; 490 ng/kg/min) were inserted into 8–10 week-old caveolin-1 knockout (*Cav1*^−/−^) mice and their C57Bl/6 J wild-type controls (WT) for a period of two weeks. Following two weeks, (**A**) systolic blood pressure was measured via tail-cuff plethysmography (n = 9–11). (**B**) The number of adhered leukocytes to excised aorta from treated mice was quantified (n = 8–10) and (**C-E**) aortic gene expression of *Ccl2*, *Icam1*, and *Il6* was determined via real-time PCR (n = 4–6). (**F–I**) Kidneys from saline- or Ang II-treated *Cav1*^−/−^ and WT mice were assessed for macrophage content. (**F**) Kidneys were sectioned and stained for CD68 (scale bar = 100 µm) and quantified based on (**G**) total kidney or (**H**) glomerular area (n = 4–5). (**I**) In parallel, kidney lysates were stained with anti-CD45 and anti-F4/80 and assessed via flow cytometry (n = 4). (**J–M**) Real-time PCR was performed to assess the gene expression of *Adgre1* (*F4/80*), *Cd68*, *Icam1*, and *Il6* (n = 4–6). All results are expressed as mean ± SEM. Data were analysed with a two-way ANOVA with Bonferroni post hoc test where *p < 0.05, **p < 0.01 and ***p < 0.001.

**Figure 6:**
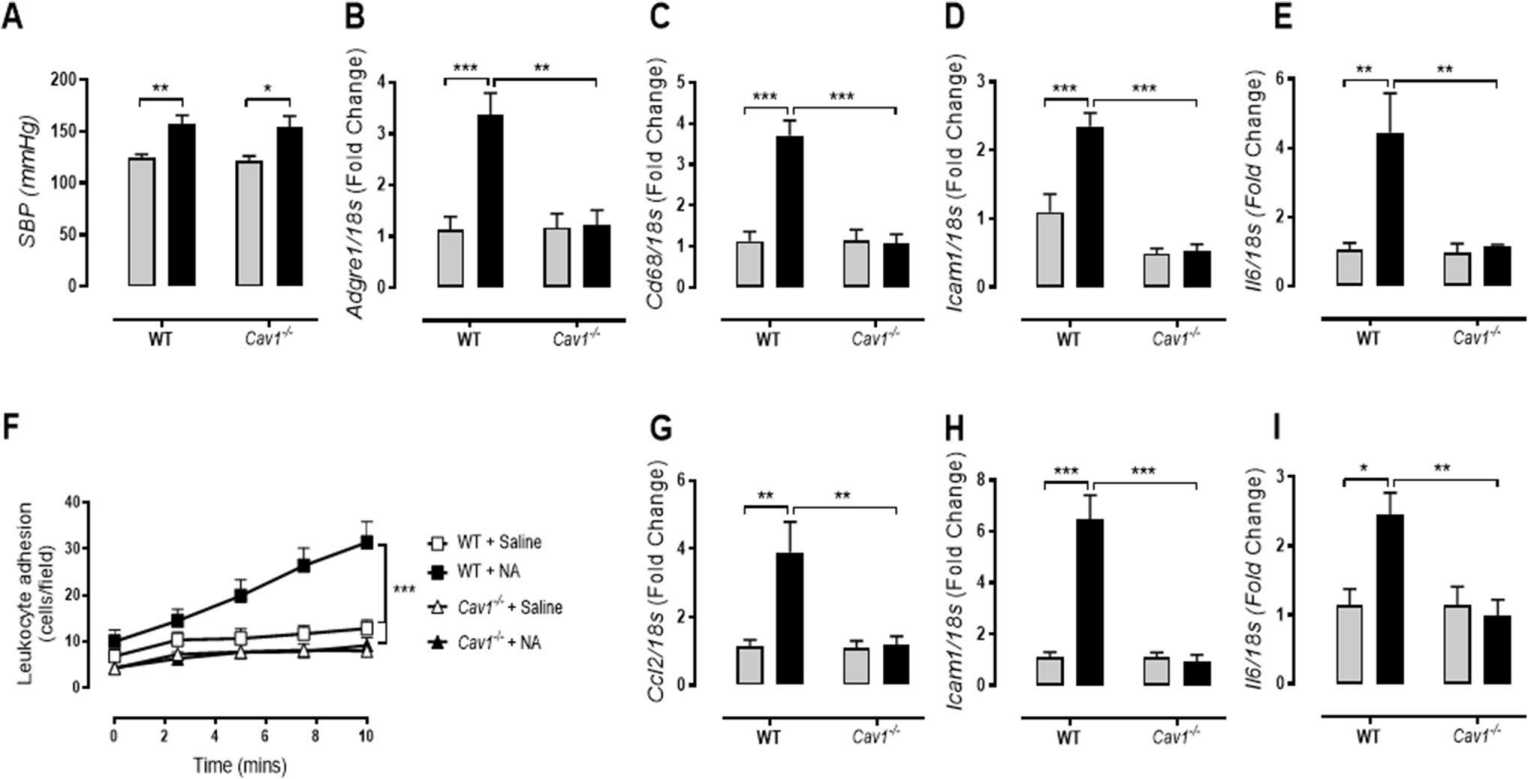
Caveolin-1 is necessary for renal and vascular inflammation in noradrenaline-induced hypertension. Minipumps containing saline (grey bars) or noradrenaline (NA; 3.8 µg/kg/min; black bars) were inserted into 8–10 week-old caveolin-1 knockout (*Cav1*^−/−^) mice and C57Bl/6 wild-type controls (WT) for a period of 2 weeks. (**A**) Systolic blood pressure was measured via tail-cuff plethysmography (n = 6–10). (**B-E**) Renal gene expression of *Adgre1*, *Cd68*, *Icam1*, and *Il6* was determined via real-time PCR (n = 4–6). Aortae were excised from saline- or NA-treated *Cav1*^−/−^ and WT mice and the number of adhered leukocytes quantified (**F**; n = 6–8). Aortic gene expression for *Ccl2*, *Icam1*, and *Il6* was also determined via real-time PCR (**G-I**; n = 5–7). All results are expressed as mean ± SEM. Data.

To examine whether the pressure effect seen in our in vitro and ex vivo models was independent of other systemic hypertensive models, we examined the effects of Ang II and noradrenaline (NA) infusion on endothelial activation and macrophage infiltration. Two-weeks of Ang II infusion induced similar increases in systolic blood pressure in both C57BL/6 J (WT) and caveolin-1 knockout (*Cav1*^−/−^) mice (Fig. 5A). The elevated systolic blood pressure in WT mice resulted in increased leukocyte adhesion (Fig. 5B) as well as elevated *Ccl2*, *Icam1*, and *Il6* gene expression (Fig. 5C-E). However, despite the elevated systolic blood pressure, *Cav1*^−/−^ mice treated with Ang II demonstrated little aortic inflammation, with significantly reduced levels of inflammatory gene expression compared to Ang II-treated WT mice (Fig. 5B-E). To determine if this effect was true in other organ pathologies associated with hypertension, we examined macrophage infiltration in the kidney. Indeed, kidneys probed for CD68 (immunohistochemistry) showed that Ang II-treated WT mice exhibited increased CD68^+^ macrophage infiltration (interstitial and glomeruli regions) (Fig. 5F-H). A similar trend was also seen in F4/80 staining of digested kidneys by flow cytometry (Fig. 5I). Inflammatory gene expression in these kidneys was also elevated (Fig. 5J-M), interestingly, this phenotype was absent in the kidneys from Ang II-treated *Cav1*^−/−^ mice (Fig. 5F-M). To determine whether this macrophage infiltration via Cav1 in vivo is caused by hypertensive stimuli other than Ang II, we treated WT and *Cav1*^−/−^ mice with a noradrenaline infusion (Fig. 6). We found that noradrenaline induced hypertension in both strains to a similar level (Fig. 6A). Similar to the Ang II treatments, noradrenaline-treated WT mice had increased renal inflammatory gene expression (Fig. 6B-E) and aortic inflammation (Fig. 6F-I). Strikingly, this response was again lost in *Cav1*^−/−^ mice suggesting that both pressure-induced endothelial inflammation and macrophage infiltration are dependent upon Cav1 expression but independent of Ang II.

## Discussion

**Figure 7:**
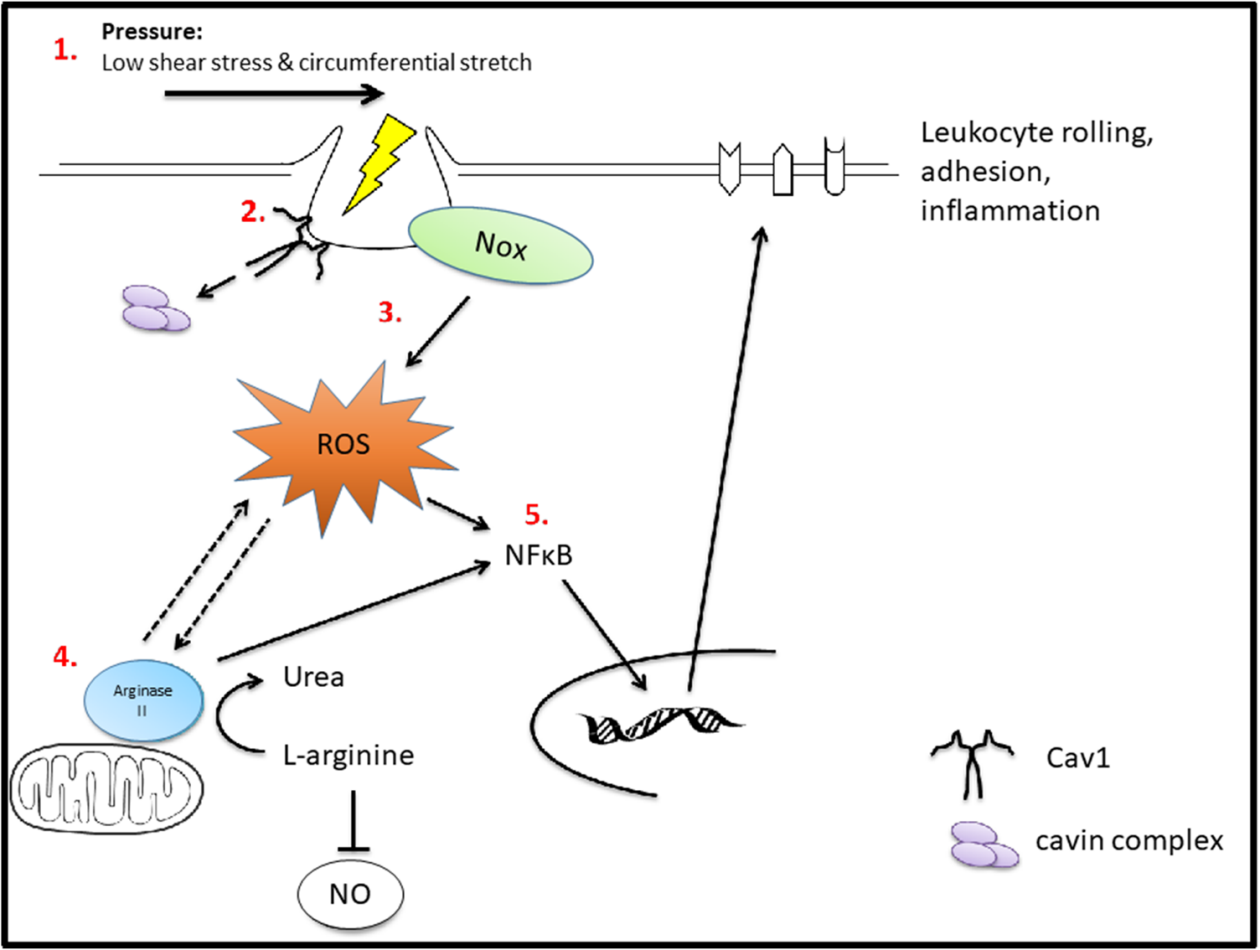
Mechanistic summary of pressure-induced inflammation. (1) Acute high intraluminal pressure exerts both low shear stress and circumferential stretch, (2) disassembling caveolae proteins (Cav1 and cavins) and causing flattening and reduction of caveolae. (3) This disassembly and/or pressure exertion leads to an increase in Nox-dependent ROS production, which in turn (4) results in an upregulation of arginase II activity and subsequently activating a feedback loop to further increase ROS production. (5) The increase in arginase II and ROS causes a translocation of NFkB into the nucleus resulting in increased gene and protein expression of adhesion molecules.

First described in 1953, caveolae are key vascular endothelial mechanotranducers that sense changes in blood flow and pressure^21^. In this study, we demonstrate that caveolae are fundamental in transducing increased intravascular pressure to a vascular inflammatory response. Specifically, we found that increased vascular pressure lead to reduced/disassembled caveolae, increased ROS activation, increased arginase II expression and NFκB translocation, and ultimately leukocyte to endothelial adhesion (Fig. 7). Surprisingly, this was found to be Ang II independent. Furthermore, we demonstrate that Cav1 (the integral membrane protein of caveolae) helps regulate pressure (Ang II or noradrenaline)-induced vascular and glomerular infiltration of macrophages. These results highlight the important role of Cav1 in pressure-induced renal and vascular inflammation.

The ability to dissect the causative mechanism between high intraluminal pressure and other neurohumoral signals (e.g. the renin-angiotensin system) that are involved in high blood pressure, has proven challenging, particularly in studying the role of pressure in the pathogenesis of vascular inflammation. In vitro studies have shed some light, demonstrating that mechanical forces exerted by pressure can directly stimulate monocyte adhesion^25^, exocytosis, and von Willebrand factor and P-selectin expression^26^. Importantly, this was an Ang II-independent effect. Indeed, our results reveal that high pressure in ex vivo vessels, in real time, directly increases leukocyte adhesion, endothelial activation, and dysfunction, in a process that appears to be Ang II-independent.

Our data are consistent with the already recognized role of caveolae as mechanosensors in other systems. In the vasculature, caveolae are abundant in endothelial cells where they house adhesion molecules and regulate eNOS activation^27^. The location of eNOS to plasmalemmal caveolae was first demonstrated by Shaul and colleagues^28^ and since then it has been established that eNOS is inactive when bound to Cav1^29^ and is activated with changes in shear stress^30^. Because of this, caveolin is commonly thought to play a role in both the progression of hypertension and atherosclerosis. While reports have been conflicting in Cav1′s role in hypertension, overall studies show *Cav1*^−/−^ mice do not demonstrate reduced changes in systemic blood pressure^31–33^ but *Cav1* knockouts may actually exhibit a pulmonary hypertensive phenotype^34^. Our study, in accordance with others, saw no change in blood pressure in Ang II- or noradrenaline-infused *Cav1*^−/−^ mice.

While there was no effect on blood pressure when *Cav1* was deleted, we did see dramatic changes with regard to vascular inflammation. The importance of Cav1 in contributing to vascular pathologies has been suggested as Cav1 is essential in transporting atherogenic LDL particles across endothelial cells^35–38^. Indeed, protection from atherosclerosis has been observed when *Cav1* is deleted in *Apoe* knockout mice through reductions in endothelial CD36 and adhesion molecules^39^. *Cav1* deletion has also been shown to reduce leukocyte adhesion in vivo^40^. The reduced plaque formation is also shown to be specifically dependent on endothelial caveolae, where re-expression of endothelial *Cav1* in the double knockout mice results in a similar atherosclerotic phenotype to control *Apoe*^−/−^^41^. In addition, overexpression of *Cav1* accelerated atherosclerosis in *Apoe*^−/−^ mice^42^.

Mechanistically, caveolae are home to an abundance of NADPH oxidase subunits^43^ and it has been proposed that Cav1 can directly regulate NADPH oxidase-induced ROS production^44^. Thus, given we saw an increase in the abundance of ROS under conditions of high pressure, it is likely that dysregulation of caveolae was an important contributor to enhanced ROS and the resulting vascular inflammation. Ex vivo, we also demonstrated diminished leukocyte adhesion with NADPH oxidase and mitochondrial ROS inhibition. Decreased bioavailability of NO is an important mechanism in both vascular inflammation and hypertension and impaired NO production may be a result of reduced eNOS activity, substrate/cofactor availability, presence of endogenous inhibitors and localization of eNOS. In hypertension, NO is also readily scavenged by NADPH oxidase to form peroxynitrite and eNOS is commonly ‘uncoupled’ and rather than producing NO, superoxide is preferably formed^45^. Further, we saw increased expression of arginase, which would be expected to compete with eNOS for L-arginine. Arginase can contribute to the progression of hypertension, and inhibiting this enzyme in spontaneously hypertensive rats does indeed reduce blood pressure and improve vascular function^46^. Furthermore, both isoforms (arginase I and II) are increased in these rats before the development of overt hypertension^19^, suggesting that arginase may play pathological roles outside of hypertension. In line with this hypothesis, inhibition of arginase has been shown to reduce plaque size in *Apoe*^−/−^ mice^47,48^. As previously demonstrated^49^, while generally not a focus in hypertension‐induced inflammation, arginase may have a more fundamental role than once thought. However, the exact mechanism by which arginase II acts is still unclear. Previous studies allude to a role of arginase as not only an early target of increased ROS^50^ but also a stimulator of ROS^51^. Furthermore, inhibition of Nox also reduces arginase activity^52^ highlighting that ROS and arginase are tightly connected. Thus, taken in context with our data, arginase II appears critical in regulating pressure-induced inflammation, supporting our previous findings that overexpression of arginase II results in endothelial dysfunction and enhanced atherosclerosis in *Apoe*^−/−^ mice^53^. Additionally, we demonstrated that NFκB is significantly increased in the nucleus of cells with acute high pressure. This is in line with previous studies demonstrating that pressure results in the degradation of IκBα and increased NFκB translocation to the nucleus^25,54^. NFκB translocation is a key step in apoptosis and inflammatory signaling pathways, propagating the upregulation of gene transcription and production of various inflammatory mediators. Although well known to be activated by LPS, inflammatory cytokines such as TNFα, and IL‐1, as well as oxidative stress^55^, little is known about how pressure or mechanical forces induce activation. Leychenko et al., (2011) have previously demonstrated that cyclical stretch of cardiomyocytes can induce NFκB signaling^56^, while Chaqour et al., (1999) showed that mechanical stretch increases platelet activation via NFκB^57^. Here we show, for the first time, that pressure‐dependent NFκB translocation is dependent on both arginase and Nox production. This is consistent with the findings of Ckless et al., (2007) who observed that arginase could regulate NFκB translocation via an NO‐dependent pathway^58^.

Moreover, our observations of reduced caveolae expression in response to pressure not only reinforce the importance of caveolae in the downstream signaling pathways seen in pressure-induced pathologies, but also relationship between cell morphology and function. Whilst we saw no evidence of caveolae internalization, we observed a trended increase in cell membrane length (data not shown), suggesting pressure-induced caveolae flattening. Similarly, in a study conducted by Sinha et al. (2011), endothelial cells exposed to a mechanical stimulus demonstrated significant reductions in the number of caveolae expressed on the cell membrane surface^24^. Importantly, they proposed this to be due to cell surface flattening as a consequence of caveolae disassembly, not internalization^24^. Indeed, HUVECs treated with MβCD showed inhibited Cav1 expression, highlighting potential caveolar disassembly^59^. In addition, they observed a significant reduction in monocyte adhesion to HUVECs treated with MβCD, suggesting an impairment in cellular mechanisms mediated in part by caveolae^59^. While the notion that caveolae flatten has been around since the 1970s ^60^ its remained largely unexplored compared to that of roles in eNOS activity, redox signaling, and lipid transport. However, more recently studies have demonstrated why caveolae flatten and the consensus is that caveolae are membrane reservoirs and flatten in response to stretching of the plasma membrane triggering cavin and caveolin disassembly^24,60–62^. It is currently unclear the exact ‘how’ caveolae flatten, however it may be as simple as the folds generated by the invaginations simply flatten in response to the increased tension. While caveolae are also known to endocytose in response to plasma membrane damage, this is not an effect from mechanical forces^61^. It is postulated that this flattening is a form of mechanoprotection and occurs to increase the cell surface area and prevent membrane rupture or cell lysis. In addition, it has been shown that *Cav1*^−/−^ mice are prone to endothelial plasma membrane rupture with increased intraocular pressure^63^ or elevated cardiac output^61^. While this may seem paradoxical to the current study, the acute effects we are demonstrating may be short-term downstream signaling for maintaining long-term cell homeostasis.

In conclusion, we provide evidence for a direct effect of raised pressure on vascular inflammation, and in turn, monocyte attachment and macrophage infiltration. These are among the major events which play a role in the unexplained relationship between high blood pressure and atherosclerosis. These data raise the possibility that therapeutic modification of caveolar mechanotransduction might be a target by which to attenuate progressive vascular remodelling and inflammation in the setting of hypertension and ischaemic cardiovascular disease.

## Materials and methods

### Animal experiments

All experiments involving animals were approved by the Alfred Medical Research and Education Precinct Animal Ethics Committee (E/1062/2011/B, E/1315/2013/B), which adhere to the National Health and Medical Research Council (NHMRC) Australian Code of Practice for the Care and Use of Animals for Scientific Purposes. The described studies were carried out in compliance with the ARRIVE guidelines. All animals were housed at the Precinct Animal Centre with a 12-h light cycle and unrestricted access to water and standard chow. Male 10 week-old Sprague Dawley rats, 8–10 week-old C57Bl/6 (wild-type, WT) and caveolin-1 knockout (*Cav1*^−/−^) mice were used in this study. WT and *Cav1*^−/−^ mice were administered with either saline, angiotensin II (Ang II; 490 ng/kg/min), or noradrenaline (NA; 3.8 µg/kg/min) for 2 weeks via osmotic minipumps (Model 2002; Alzet, Cupertino, CA, USA) to induce hypertension. In a separate cohort, WT mice were administered with either saline or Ang II via osmotic minipumps (Model 2004; Alzet, Cupertino, CA, USA) to induce hypertension over a 4-week period. During the final 2 weeks, mice received either subcutaneous injections of either 2-hydroxypropyl-β-cyclodextrin (2HPβCD; 2 g/kg body weight; Merck KGaA, Darmstadt, Hesse, Germany) or saline twice a week.

### Tail-cuff plethysmography

Systolic blood pressure was assessed in conscious WT and *Cav1*^−/−^ mice using non-invasive tail-cuff plethysmography as previously described^64^. Briefly, mice were gently placed on a heated platform (37.8 °C) to facilitate blood flow to their tails, while being restrained in individual chambers to limit movement. The tail of each mouse was inserted through a tail cuff, which automatically inflates for the occlusion of blood flow. The MC4000 Blood Pressure Analysis System (Hatteras Instruments, Cary, North Carolina, USA) records the pulse during the deflation of the cuff displaying SBP readings in mmHg. Five preliminary measurement cycles were conducted followed by ten measurement cycles, which were averaged and recorded. At the end of the procedure, the mice were returned to their cages. Mice were acclimatised to the procedure in the two weeks leading up to the measurements taken.

### Ex vivo pressurised shear flow leukocyte adhesion assay

To determine the effect of pressure on inflammation an ex vivo vessel chamber was used as previously described^65^. In brief, isolated rat carotid arteries (RCA) were mounted and pressurised at either 0, 60, 80 or 120 mmHg for 1 h (Living Systems Instrumentation, Burlington, VT, USA). In some instances, RCA pressurised to 120 mmHg were subjected to either allopurinol (100 μM), apocynin (3 μM), BEC ([S-(2-boroethyl)-L-cysteine], 100 μM), candesartan (100 nM), cyclosporin A (2 μM), methyl-β-cyclodextrin, (MβCD; 10 mM), or miconazole (30 μM). Pressurised RCA or mouse aortae were then perfused with human whole blood (100 µl/min) labelled with Vybrant DiI Cell-Labelling Solution (1:1000; Thermo Fisher Scientific, Waltham, MA, USA) -to fluorescently tag leukocytes. Whole blood was obtained from healthy donors following informed written consent and all experiments were approved by the Alfred Hospital Ethics Committee (397/09) and carried out in accordance with relevant guidelines and regulations. Of note, as with using FBS for human and mouse cell experiments, there is always a chance for some cross reactivity and an immune response, therefore control treatments were always tested on the same day (same whole blood) to ensure background levels were minimal. Leukocyte adhesion was imaged using a Zeiss SteREO Discovery V.20 fluorescent microscope (Zeiss, Oberkochen, Baden-Württemberg, Germany).

### Ex vivo shear flow leukocyte adhesion studies

The shear stress exerted on vessels was mathematically estimated using Hagen-Poiseuille’s equation: τ = 4ηQ/πr^3^; τ = shear stress (dyne/cm^2^); η = viscosity (poise); Q = flow (ml/sec); and r = radius (cm). Viscosity of the labeled whole blood was assumed to be at 0.035 poise. To determine the radius of the vessels, snapshot images of carotids pressurised to 120 mmHg were acquired using the Zeiss Axiovision software at 160 × magnification. The radius of the arteries was determined using a scale captured at the same magnification. At the standard flow rate (100 µl/min) shear stress was calculated at 1.67 dyne/cm^2^, equivalent to low shear stress (LSS). The vessels were subjected to LSS during incubation and perfusion of the blood. To simulate high shear stress (HSS) the flow was increased to 1 ml/min (16.7 dyne/cm^2^) during incubation and perfusion of the blood.

### In vitro pressurised cell chamber

A monolayer of HUVECs (Lonza, Muenchensteinerstrasse, Basel, Switzerland) or transformed H5V cells cultured on 25 mm round glass coverslips (Warner Instruments, Hamden, CT, USA) coated with Collagen I (BD Biosciences, San Jose, CA, USA). Once confluent they were placed in a custom stainless-steel flow-through chamber (Penn-Century, Inc., Wyndmoor, PA, USA). The chamber was filled with Krebs modified buffer (NaCl 119, KCl 4.7, MgSO4•7H2O 1.17, NaHCO3 25, KH2PO4 1.18, CaCl2 2.5, glucose 11 and EDTA 0.03 mmol/L) and maintained at 37 °C and physiological pH by infusing carbogen gas (95% O_2_; 5% CO_2_). Cells were pressurised (Living Systems Instrumentation, Burlington, VT, USA) for 1 h and underwent either RNA isolation, fluorescent microscopy or measurement of ROS.

### Gene Expression

RNA was isolated using TRIzol (Thermo Fisher Scientific, Waltham, MA, USA), DNase treated as previously described^66^, and then reverse transcribed into cDNA. Real-time PCR was performed using SYBR (Roche, Basel, Switzerland). 18 s or RpIp1 were used as housekeeping genes and fold change was calculated by the ∆∆CT. Primers used for real-time PCR: human 18S: 5′-GCCGCTAGAGGTGAAATTCTTG-3′ AND 5′-CATTCTTGGCAAATGCTTTCG-3′; human ICAM1: 5′-GCTGGAGCTGTTTGAGAACAC-3′ and 5′-CAAGTTGTGGGGGAGTCG-3′; human CCL2: 5′-TTCTGTGCCTGCTGCTCAT-3′ and 5′-GGGGCATTGATTGCATCT-3′; rat RpIp1: 5′-CTGGTGGTCCTGCTCCAT-3′ and 5′-TGTCATCCTCGGATTCTTCA-3′; rat Icam1: 5′-GCAGACCACTGTGCTTTGAG-3′ and 5′-TCCAGCTCCACTCGCTCT-3′; rat Ccl2: 5′-AGCATCCACGTGCTGTCTC-3′ and 5′-ATCATCTTGCCAGTGAATGAG-3′; rat Nos3: 5′-TGACCCTCACCGATACAACA-3′ and 5′-ATGAGGTTGTCCGGGTGTC-3′; rat ArgI: 5′-CCGCAGCATTAAGGAAAGC-3′ and 5′-CCCGTGGTCTCTCACATTG-3′; rat ArgII: 5′-CTAGTGAAGCTGCGAACGTG-3′ and 5′-GCCCTAGCAGAAGCAGCTC-3′; mouse 18 s: 5′-TTGACGGAAGGGCACCACCAG-3′ and 5′-GCACCACCACCCACGGAATCG-3′; mouse Icam1: 5′-CCCACGCTACCTCTGCTC-3′ and 5′-GATGGATACCTGAGCATCACC-3′; mouse Ccl2: 5′-TCTTACCTGTGCGCTGTGAC-3′ and 5′-ACTGGATCTTCAGGGAATGAGT-3′; mouse Il6: 5′-CTTCCATCCAGTTGCCTTCTTG-3′ and 5′-AATTAAGCCTCCGACTTGTGAAG-3′; mouse Cd68: 5′-ACTTCGGGCCATGTTTCTCT-3′ and 5′-GCTGGTAGGTTGATTGTCGT-3′; and mouse Adgre1: 5′-CTTTGGCTATGGGCTTCCAGTCC-3′ and 5′-GCAAGGAGGACAGAGTTTATCGTG-3’.

### Real time ROS detection in pressurised endothelial cells

HUVECs were seeded in the pressurised chamber as described above. Using a fluorescent upright Olympus microscope coupled to a digital camera, ROS was determined using either 2′,7′-Dichlorodihydrofluorescein diacetate (DCFHDA; Merck KGaA, Darmstadt, Hesse, Germany) or the more sensitive H_2_O_2_ indicator Peroxyfluor-6 acetoxymethyl ester (PF6-AM, a gift from Christopher Chang^67^). Cells were perfused with Krebs modified buffer (1 ml/min) with either DCFHDA (5 µM) or PF6-AM (5 µM) for 30–60 min for stabilisation. Following stabilisation, pressure (0, 60 or120 mmHg) was applied for 1 h followed by 30 min of no pressure. Images were acquired every 10 min using the Zeiss Axiovision software (Zeiss, Oberkochen, Baden-Württemberg, Germany) and stored for offline analysis. Fluorescence intensity was determined using ImageJ 1.47 g and data was expressed as mean change in intensity compared to the average fluorescence measured during the stabilisation period.

### Electron microscopy preparation of rat carotids

Pressurised RCA were fixed with 100 mM cacodylate buffer pH 7.4 containing 2.5% glutaraldehyde. 1 mm thick segments were washed in 100 mM of fresh cacodylate buffer and then stored at 4 °C. Samples were osmicated with 1% osmium tetroxide in 100 mM cacodylate buffer and then Epon-embedded and sectioned using a Rechert Ultracut S ultramicrotome. The Epon block was trimmed and 70–90 nm sections were cut using a Diatome 45° diamond knife. Sections were collected on 200 square mesh Copper-Paladium grids and stained with 2% uranyl acetate, washed with distilled water and stained with Reynold’s Lead Citrate solution for 5 min and washed again. Sections were left to dry thoroughly at room temperature.

### Imaging and quantifying endothelial caveolae

Using a transmission electron microscope (Hitachi H7500; Monash Micro Imaging; Monash University, Clayton, Australia) images were captured at 60,000 × magnification. Caveolae were defined as 60–100 nm invaginations of the luminal membrane. Two fields from each cell were counted using ImageJ software using an average of a minimum 25 cells per vessel. Length of the plasma membrane was also quantified and results expressed as the number of caveolae/length of luminal membrane (μm).

### Intracellular NFĸB staining

HUVECs were pressurised to 0, 120 mmHg alone or 120 mmHg with inhibitors apocynin (3 µM), BEC 100 µM), or SN50 (20 µM) for 30 min. and then fixed in 4% formaldehyde and stained as previously described^68^. Briefly, cells were blocked for 1 h in PBS (0.3% TritonX100, 5% normal rabbit serum) and then incubated overnight at 4 °C with primary antibody (1:50; NFĸB; Cell Signaling Technology, Danvers, MA, USA) in PBS (0.1% tween20, 0.1% BSA). Cells were washed with PBS and incubated for 1 h at room temperature with the secondary antibody alexa fluor 546 (1:500; Thermo Fisher Scientific, Waltham, MA, USA) in PBS (0.1% tween 20, 0.1% BSA). Cells were then washed, counterstained and mounted in DAPI mounting medium (5 µl; Thermo Fisher Scientific, Waltham, MA, USA) and z-stack imaged using a Nikon AR1 confocal microscope (Monash Micro Imaging, Clayton, VIC, Australia).

### Ruthenium red stained pressurised carotids

RCA were pressurised at 0, 80 and 120 mmHg for 1 h and fixed in 100 mM cacodylate buffer ph 7.4 containing 2.5% glutaraldehyde and 1 mg/ml of ruthenium red. Samples were dehydrated, sectioned, and imaged with a transmission electron microscope.

### Fluorescence staining of Cav1 and cavin-1

Following 1 h of continuous pressure, rat carotids were perfused with paraformaldehyde (4%) under pressure. Vessels were then removed from the chamber and cut into two–three segments and frozen in optimal cutting temperature embedding compound in liquid nitrogen and stored at −80 °C. The ends of the vessels attached to the cannula were discarded. Using a cryostat (Zeiss 102,717 Microm HM 550) 6 μm sections were cut and 3 Sects. (30 μm apart) were placed on the slide with 10 slides per vessel. Slides were soaked in PBS and once dry, paraffin outlines were drawn around each section. Blocking buffer (0.2% BSA, 0.2% FSG in PBS) was added to each section and incubated at room temperature for 15 min. One section from each slide was aspirated and the primary antibody mix (30 μl) consisting of Cav1 (1:200, BD, #610,407), Cavin1 (1:150, made in house) and the blocking solution was added. After incubating overnight at 4 °C, slides were washed 3 times for 5 min with PBS. Secondary antibody mix (30 μl) consisting of alexa fluor anti-mouse 488 (1:500; Thermo Fisher Scientific, Waltham, MA, USA), alexa fluor anti-rabbit 555 (1:500; Thermo Fisher Scientific, Waltham, MA, USA) and blocking solution was added to all sections. Following 30 min incubation at room temperature, sections were washed 3 times for 5 min in PBS. All slides were aspirated and DAPI stain (1:500; Thermo Fisher Scientific, Waltham, MA, USA) was added and incubated for 10 min. Slides were then rinsed in distilled water and coverslips were mounted. Slides were dried overnight at room temperature and then stored at 4 °C before imaging.

### CD68 staining

6 µm paraffin-embedded kidney sections were dewaxed and washed with PBS twice for 5 min followed by an incubation in 3% hydrogen peroxide in methanol for 20 min. Sections were washed with PBS (2 × 5 min) again and blocked with 10% normal goat serum (NGS) for 30 min and incubated with avidin blocking solution (Vector Laboratories, Burlingame, CA, USA) for 15 min. After a rinse in PBS, sections were incubated with Biotin blocking solution (Vector Laboratories, Burlingame, CA, USA) for 15 min and rinsed again with PBS. Sections were incubated overnight at 4 °C with either the CD68 primary antibody (1:200; rat anti-mouse; Bio-Rad Laboratories, Hercules, CA, USA) in 5% NGS or 5% NGS only (negative control). Sections were washed with PBS (2 × 5 min) and incubated with the secondary antibody (1:100; mouse anti-rat; BD Biosciences, San Jose, CA, USA) in 5% NGS for 30 min. After a wash in PBS (2 × 5 min) the ABC complex (Vector Laboratories, Burlingame, CA, USA) was added for 30 min followed by further washes in PBS (2 × 5 min). The DAB (3,3′-Diaminobenzidine) mix (Vector Laboratories, Burlingame, CA, USA) was added until the sections turned brown. Sections were then washed in ultrapure water (2 × 5 min) and stained with Mayer’s Haematoxylin (45 s). Residual stain was removed by a rinse in running tap water until clear, 4 min in Scott’s tap water and another rinse in tap water. Sections were dehydrated with 95% ethanol (3 min) followed by three changes in 100% ethanol (3 min each) and cleared in two changes of xylene (5 min each). Sections were then air dried and mounted with Depex Mounting Medium and dried overnight. Sections were imaged at 10 × and 40 × using the FSX100 light microscope (Olympus, Shinjuku, Tokyo, Japan).

### F4/80 staining via flow cytometry

Kidneys were perfused with PBS to remove excess blood, followed by enzymatic digestion in Liberase (Merck KGaA, Darmstadt, Hesse, Germany) for 4 min at 37 °C for 45 min. Following enzymatic digestion, samples were filtered through 70 µm filters and single cells were washed with PBS. Single cell pellets were acquired via centrifugation and pellets were resuspended in lysis buffer to eliminate red blood cells. Following this, cells were stained with a viability marker (1:1000; Thermo Fisher Scientific, Waltham, MA, USA) to gate for live cells, CD45 (1:200; Anti-mouse CD45; Biolegend, San Diego, CA, USA) to gate for total leukocytes, and F4/80 (1:200; Anti-mouse F4/80; eBioscience, San Diego, CA, USA) for macrophages. Samples were run on the LSR Fortessa X-20 (BD Biosciences, San Jose, CA, USA) and data was analysed using FlowJo software (FlowJo, Ashland, OR, USA).

### Statistics

Statistical significance was determined using a 2-tailed Student’s t-test, one-way ANOVA, or two-way ANOVA, followed by post hoc analysis. A *P* value of less than 0.05 was deemed statistically significant. All data are expressed as mean ± standard error of the mean (SEM), unless otherwise noted, and statistical analysis was carried out using Graphpad Prism 6.0 software (GraphPad Software, San Diego, CA, USA).

## Supplementary Information

Supplementary Information

## Abbreviations

2HPβCD: 2-Hydroxypropyl-β-cyclodextrin
Ang II: Angiotensin II
BEC: S-(2-boroethyl)-L-cysteine
Cav1: Caveolin-1
CCL2: CC chemokine ligand 2
DAB: 3,3′-Diaminobenzidine
DCFHDA: 2′,7′-Dichlorodihydrofluorescein diacetate
HUVECs: Human umbilical vein endothelial cells
HSS: High shear stress
ICAM-1: Intracellular adhesion molecule 1
LSS: Low shear stress
MCP1: Monocyte chemoattractant protein 1
NA: Noradrenaline
NFĸB: Nuclear factor κB
NGS: Normal goat serum
PF6-AM: Peroxyfluor-6 acetoxymethyl ester
RCA: Rat carotid artery
