# Supplementary material for: High intraluminal pressure promotes vascular inflammation via caveolin-1
Source: Sci Rep. 2021 Mar 15;11:5894. doi: 10.1038/s41598-021-85476-z (PMC7960707; doi:10.1038/s41598-021-85476-z)
Supplement: Supplementary file 1 — Supplementary Information [file 41598_2021_85476_MOESM1_ESM.pdf]

High intraluminal pressure promotes vascular inflammation via caveolin-1

Danielle L. Michell (PhD)[1,2,8], Waled A. Shihata (PhD)[1,2,3,8*], Karen L. Andrews (PhD)[1,3,4], Nurul Aisha Zainal Abidin (Ms)[3,4], Ann-Maree Jefferis (BSc)[4], Amanda K. Sampson (PhD)[1], Natalie G. Lumsden (PhD)[1], Olivier Huet (MD, PhD)[1], Marie-Odile Parat (PhD)[6], Garry L. Jennings (MD)[1], Robert G. Parton (PhD)[5], Kevin J. Woollard (PhD)[1,7], David M. Kaye (MD, PhD)[1], Jaye P. F. Chin-Dusting (PhD)[1,2,3,4], and Andrew J. Murphy (PhD)[1]

[1] Baker Heart and Diabetes Institute, Victoria, Australia

[2] Department of Medicine, Monash University, Victoria, Australia

[3] Cardiovascular Disease Program, Biomedicine Discovery Institute, Monash University

[4] Department of Pharmacology, Monash University, Victoria, Australia

[5] Institute for Molecular Bioscience and Centre for Microscopy and Microanalysis, University of Queensland, Queensland, Australia

[6] School of Pharmacy, University of Queensland, Queensland, Australia

[7] Centre for Inflammatory Disease, Department of Immunology and Inflammation, Imperial College London, London, United Kingdom

[8]co-first author

[*]corresponding author

Dr Waled A. Shihata
Heart Failure Research Group
Baker Heart and Diabetes Institute,
75 Commercial Rd, Melbourne 3004, AUSTRALIA
T +61 3 8531 1486
E waled.shihata@baker.edu.au

Running title: Cav1 influences pressure-induced inflammation

SUPPLEMENTARY FIGURES AND FIGURE LEGENDS

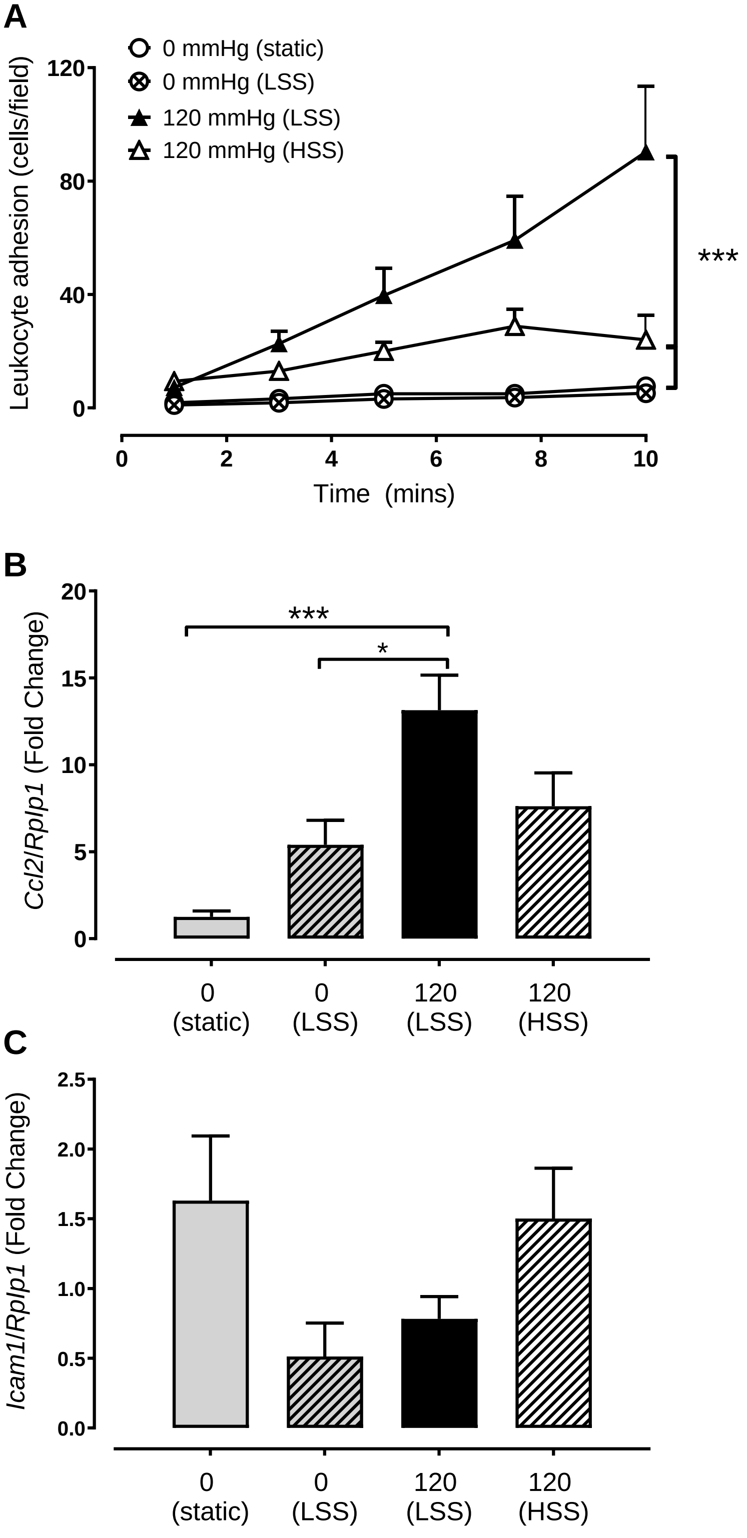

Figure S1. Isolated rat carotid arteries were subjected to no pressure (0 mmHg) without flow (static), or with low shear stress (LSS; 1.67 dyne/cm^2), or high pressure (120 mmHg) with LSS or high shear stress (HSS; 16.67 dyne/cm^2), n=5-6. (A) Quantification of leukocyte adhesion per field under different shear flow. (B-C) Gene expression of *Ccl2* and *Icam1* in shear flowed carotid arteries by real-time PCR. All results are expressed as mean ± SEM. Data were analysed with a one-way (B-C) or two-way ANOVA (A) with Bonferroni post hoc test where *p<0.05 and ***p<0.001.

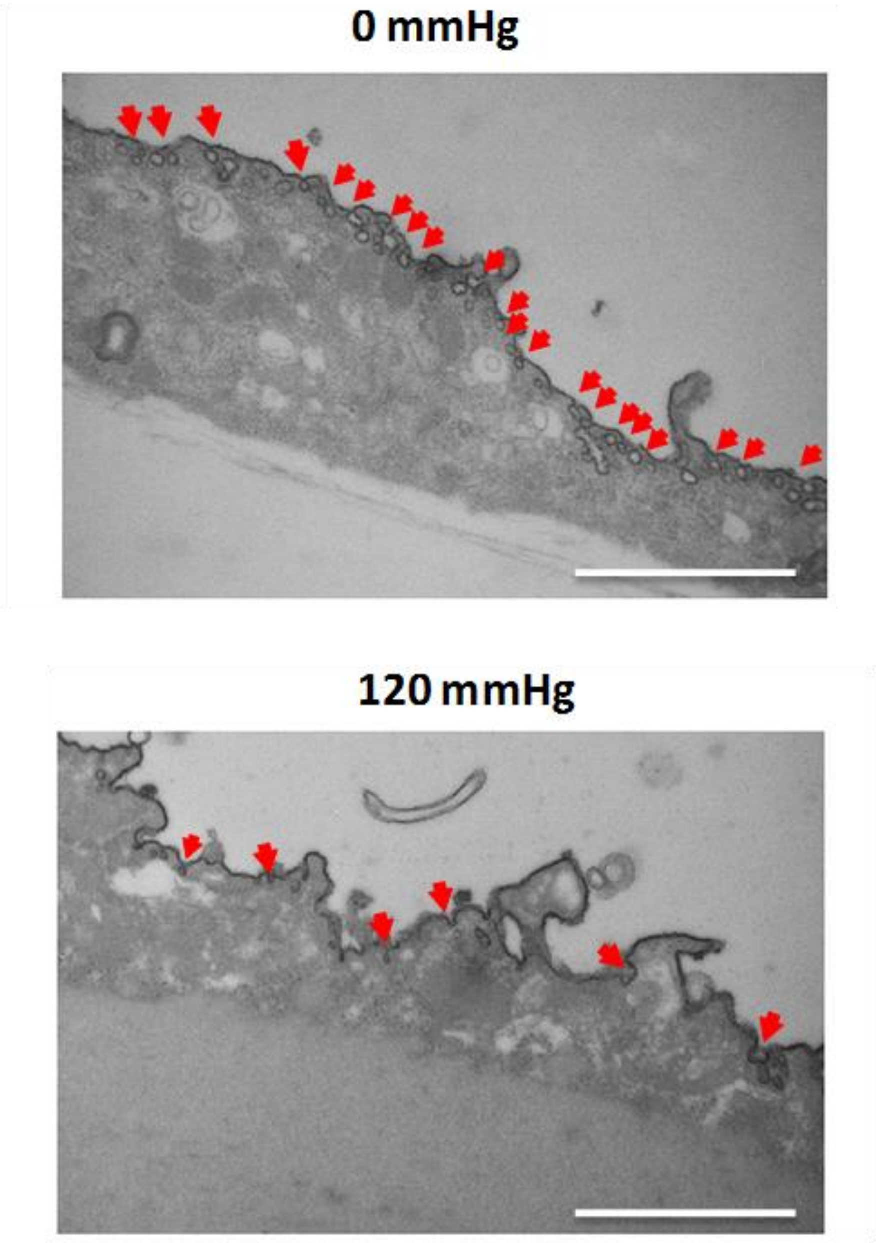

Figure S2. Ruthenium red stained caveolae in pressurised carotid arteries. Representative images of caveolae (red arrows) in rat carotid arteries (0 and 120 mmHg) stained in the presence of the membrane marker ruthenium red. Scale bar=1000 nm.

Blot 1

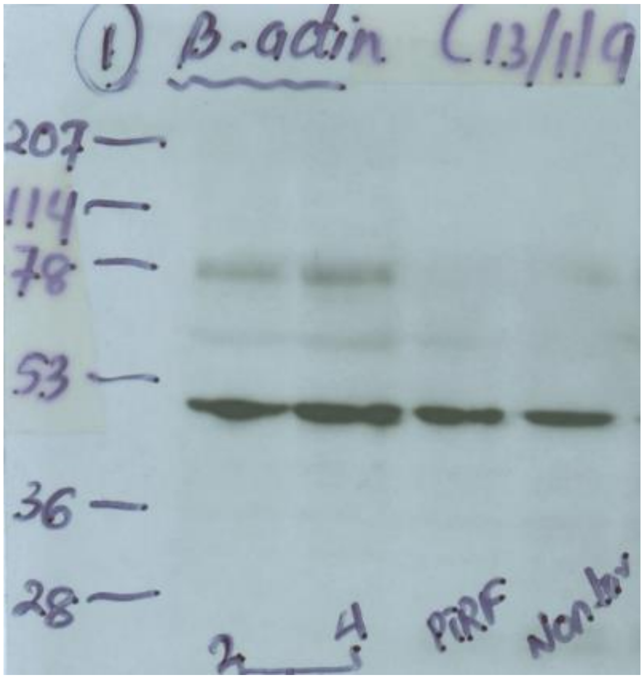

Blot 2

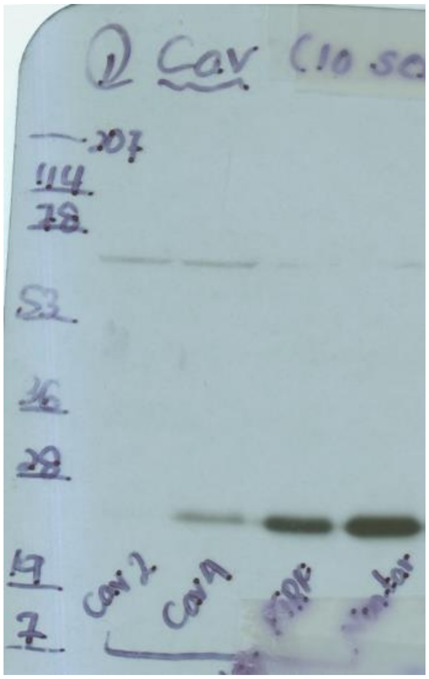

Figure S3. Full blots from Figure 4a. Four cell types were tested to confirm specific Cav1 knockdown. shRNA was introduced using a lentivirus where two distinct sequences (#2 and #4) were used to target the gene of interest (Cav1). The cell types were as follows:

- H5V shCav sequence 2 (indicated as 2 and Cav 2 on blots 1 and 2, respectively)
- H5V shCav sequence 4 (indicated as 4 and Cav 4 on blots 1 and 2, respectively)
- H5V PTRF downregulated (indicated as PTRF on blots 1 and 2)
- H5V shScr (indicated as Non-tar on blots 1 and 2)

Blot 1 probed for the housekeeper β-actin. Blot 2 probed for Cav1. Sequence 2 and Non-tar cells were used for all cell culture studies utilising H5V cells (H5V shCav and shScr, respectively). As such, we have only included the relevant bands in Figure 4a as proof of successful transfection and that the cells used had Cav1 knockdown.
